# Appraisal of current technologies for the study of genetic alterations in hematologic malignancies with a focus on chromosome analysis and structural variants

**DOI:** 10.1515/medgen-2024-2001

**Published:** 2024-03-06

**Authors:** Itziar Salaverria, Reiner Siebert, Krzysztof Mrózek

**Affiliations:** The Ohio State University Comprehensive Cancer Center, Clara D. Bloomfield Center for Leukemia Outcomes Research Columbus USA; Institut d’Investigacions Biomèdiques August Pi i Sunyer Barcelona Spain; Ulm University Medical Center, Ulm University Institute of Human Genetics Albert-Einstein-Allee 11 89081 Ulm Germany

**Keywords:** chromosome analysis, fluorescence *in situ* hybridization, comparative genomic hybridization, optical genome mapping, next-generation sequencing

## Abstract

During the last five decades, chromosome analysis identified recurring translocations and inversions in leukemias and lymphomas, which led to cloning of genes at the breakpoints that contribute to oncogenesis. Such molecular cytogenetic methods as fluorescence *in situ* hybridization (FISH), copy number (CN) arrays or optical genome mapping (OGM) have augmented standard chromosome analysis. The use of both cytogenetic and molecular methods, such as reverse transcription-polymerase chain reaction (RT-PCR) and next generation sequencing (NGS), including whole-genome sequencing (WGS), discloses alterations that not only delineate separate WHO disease entities but also constitute independent prognostic factors, whose use in the clinic improves management of patients with hematologic neoplasms.

## Introduction

Recurrent chromosome aberrations, their molecular equivalents and other gene mutations have been included in the 5th edition of the WHO Classification of Haematolymphoid Tumors (WHO-HAEM5) [1, 2] as well as in other classifications of leukemias and lymphomas. [3, 4] Together with morphology, immunophenotype and clinical features, these genetic alterations are used to define distinct disease entities with unique patterns of responses to treatment, [1–4] and help in prognostication, therapy selection and evaluation of response to therapy. [5–8] Consequently, cytogenetic analyses are an obligatory part of the diagnostic work-up for patients with acute myeloid leukemia (AML), myelodysplastic neoplasms (MDS), acute lymphoblastic leukemia (ALL) and chronic myeloid leukemia (CML), according to recommendations of the National Comprehensive Cancer Network (NCCN) Clinical Practice Guidelines in Oncology [9–11] and the European LeukemiaNet. [12, 13] Herein, we review briefly the current methodologies for the detection of recurring genetic alterations that play a role in the clinical management of patients diagnosed with hematologic malignancies. Notably, the WHO-HAEM5 does not favor or recommend any specific technology for the detection of genetic alterations. This is in part due to the fact, that different technologies can be applied to detect the same aberration, that not all technologies are available in every place and that future developments will see upcoming methods that might substitute or complement others.

## Chromosome analysis

Chromosome analysis is a genome-wide assay capable of revealing all clonal, microscopically detectable abnormalities present in leukemic cells, albeit with a rather low resolution estimated to be 5–10 Mb. Chromosome analysis evaluates metaphase chromosomes obtained from viable, dividing cells from bone marrow, blood, lymphoid tissue, or other tumor-containing tissue using staining (banding) techniques. Of the main banding techniques, namely the Giemsa (G), reverse (R), quinacrine (Q) and centromeric (C) banding, most laboratories routinely use G-banding. C-banding is useful for more precise characterization of abnormalities of pericentromeric heterochromatin regions, whereas Q-banding is especially suitable to identify the Y chromosome in both metaphases and interphase nuclei. R-banding yields a banding pattern opposite to G-banding, with dark bands in G-banded chromosomes staining light with R-banding, and vice versa. R-banding is useful for identifying deletions or translocations involving distal regions of chromosomes, which harbor such genes as *BCL6*, *MYC* or *IGH*, and the late-replicating, inactive X chromosome.

To obtain chromosome preparations of good quality, sample processing should be individualized because specimens from patients with different hematologic disorders can have specific culture requirements. For example, B lymphoblastic leukemia/lymphoma (B-ALL/LBL) or T lymphoblastic leukemia/lymphoma (T-ALL/LBL) specimens that have a high mitotic index can grow in direct culture for 1 to 6 hours, whereas most hematologic neoplasms require short-term, unstimulated culture (24 to 48 hours). Stimulation with mitogens (e.g., DSP30 or CpG-oligonucleotide/interleukin 2) is necessary in chronic lymphocytic leukemia (CLL) or in T-cell leukemias (with PHA). [14]

Although chromosome analysis is a powerful tool for detection of somatic aberrations at a single-cell level that allows assessment of tumor heterogeneity, it can be time-consuming, technically demanding, and requires dividing cells to obtain metaphases. In many hematologic disorders, particularly lymphomas, the mitotic index can be low and the quality of metaphases poor. Karyotypes of many advanced lymphoid tumors are highly complex and cannot be entirely resolved by karyotyping. Moreover, chromosome analysis is unable to distinguish between molecularly distinct rearrangements that appear to be identical. For example, the t(14;18)(q32;q21) occurs in both follicular lymphoma (FL) and extranodal marginal zone lymphoma (EMZL) of mucosa-associated lymphoid tissue (MALT), but the deregulated genes at 18q21 are different, resulting in *IGH*::*BCL2* fusion in FL and *IGH*::*MALT1* in EMZL. It is important to differentiate between these translocations since each is associated with a distinct histologic subtype. Another shortcoming of chromosome analysis is its inability to detect cryptic translocations involving telomeric regions, such as the t(5;11)(q35;p15.5)/*NUP98*::*NSD1* in AML or t(6;14)(p25;q32)/*IGH*::*IRF4* found in multiple myeloma and in large B-cell lymphoma with *IRF4* rearrangement. [15]

## Molecular cytogenetic methods

Molecular cytogenetic methods can partially overcome the aforesaid limitations by being capable of analyzing non-dividing cells with increased resolution. The first such method was fluorescence *in situ* hybridization (FISH), followed by spectral karyotyping (SKY), multicolor FISH (M-FISH), comparative genomic hybridization (CGH) and single-nucleotide polymorphism (SNP) arrays. The applications, pros and cons of chromosome analysis and molecular cytogenetic techniques are provided in Table 1.

## FISH

In FISH assay, fluorescently labeled DNA probes are hybridized to interphase nuclei or metaphase chromosomes. FISH can also be applied to air-dried bone marrow or blood smears, fresh tumor touch prints, frozen or formalin-fixed paraffin-embedded (FFPE) tissue sections, G-banded slides, or nuclear isolates from fresh or fixed tissues.

There are large numbers of FISH probes targeting specific loci or the entire chromosome available. Probes routinely used in the analysis of hematologic malignancies include chromosome-specific enumerator (i.e., mostly centromeric) probes, locus- or gene-specific probes, whole chromosome painting probes, arm-specific sequence probes, and telomeric probes.

Chromosome-specific centromeric probes are derived from the highly repetitive, mostly alpha-satellite DNA sequences located within the centromeres. Because the target size is several hundred kilobases (kb) in length, the probes generate bright signals and are easy to evaluate in both metaphases and interphase nuclei. Centromeric probes are useful in identifying numerical abnormalities (aneuploidy), dicentric chromosomes, and the origin of marker chromosomes. Clinically important aberrations such as trisomy 12 in CLL, monosomy 7 in AML, and high hyperdiploidy in ALL—all of which can be detected at a lower frequency by karyotyping because of low mitotic index or poor chromosome morphology—are subject of routine evaluation by FISH. Also, probes specific for chromosomes Y and X are used for monitoring engraftment in sex-mismatched allogeneic hematopoietic stem-cell transplantation.

The whole chromosome painting probes or arm-specific sequence probes use mixtures of fluorescently labeled DNA sequences derived from the entire length of the specific chromosome or one of its arms. [16] These probes can only be applied to metaphase cells because in interphase cells, they generate large and diffuse signals. They are helpful in characterizing complex rearrangements and marker chromosomes. However, cryptic rearrangements affecting terminal regions may remain undetected, because the repetitive DNA sequences within these regions are not covered. For detection of these cryptic rearrangements, chromosome-specific telomeric or subtelomeric probes derived from DNA sequences located at the telomeres are most suitable.

Gene-specific or locus-specific probes are derived from unique DNA sequences or loci within the chromosome. Even using banding techniques on highly stretched (prometaphase) chromosomes, the smallest detectable chromosome abnormality is 2000–3000 kb, whereas gene- or locus-specific probes can consistently detect segments as small as 0.1 Mb. [16] Thus, these probes are widely used in both clinical and basic research. Locus-specific probes have been useful in gene mapping and characterizing structural rearrangements, amplifications, and origin of marker chromosomes. In lymphoid malignancies, locus- or gene-specific probes have also been effective in delineating minimal regions of deletion (e.g., 6q21-23, 11q22, and 13q14) and in demonstrating monoallelic losses of loci containing the *TP53* and *RB1* genes.

**Table 1:** Comparison of chromosome analysis with molecular cytogenetic techniques

**Feature**	**Chromosome analysis**	**SKY/ M-FISH**	**FISH**	**CGH**	**CGH array**	**SNP array**	**OGM**
Resolution	>5 Mb	>2 Mb	50 kb	3–10 Mb	3 kb; 1M Agilent 1M array	10–20 kb SNP6	
**Identification**							
Balanced translocations	Yes	Yes	Yes*	No	No	No	Yes
Unbalanced translocations	Yes	Yes	Yes*	?	?	?	Yes
Structural rearrangements within a single chromosome	Yes	Sometimes	Yes*	No	No	No	Yes
Origin of marker chromosome	No	Yes	Yes^†^	?	?	?	?
Copy number changes	Yes	Yes	Yes	Yes	Yes	Yes	Yes
Deletions <10 Mb	Sometimes	Sometimes	Yes	No	Yes	Yes	Yes
Allelic loss	No	No	Yes	No	No	Yes	Yes
Copy number neutral LOH	No	No	No	No	No	Yes	No
High-level amplification	Sometimes^‡^	Sometimes^‡^	Yes*	Yes	Yes	Yes	Yes
Subtelomeric rearrangements	No	No	Yes*	No	No	No	Yes
Resolves complex and cryptic chromosomal alterations	No	Yes	Yes*	No	No	No	Yes
**Pros and Cons**							
Requires specifically labeled probes	No	Yes	Yes	Yes	No	No	No
Requires prior knowledge of DNA sequences of the aberration	No	No	Yes	No	No	No	No
Scans the entire genome	Yes	Yes	No	Yes	Yes	Yes	Yes
Identifies tumor heterogeneity	Yes	Yes	Yes	No	Yes	Yes	Yes
Requires viable cells	Yes	Yes	No	No	No	No	Optimally yes
Requires tumor metaphase spreads	Yes	Yes	No	No	No	No	No
Applicable to interphase nuclei and non-dividing cells	No	No	Yes	No	No	No	No
Applicable to DNA extracted from archived tissue (FFPE)	No	No	No	Yes	Yes	No	No
Labor-intensive	Yes	Yes	No	No	No	No	No
Interpretation highly dependent on experience and knowledge	Yes	Yes	Yes	Yes	No	No	No
Expensive for small diagnostic laboratories	No	Yes	No	Yes	Yes	Yes	Yes
Applicable and cost-effective as a routine screening method	Yes	No	Yes	No	No	No	Limited
Turnaround time (days)	3–10	2–7	2–7	2–3	3–4	3–4	4–6

Modified from Salaverria I, Siebert R, Mrózek K (2017) Important chromosomal aberrations in hematologic neoplasms and key techniques to diagnose them. In: Jaffe ES, Arber DA, Campo E, et al. editors. Hematopathology, 2nd edition. Philadelphia, PA: Elsevier, 105–128

*Only with appropriately designed probes.

^†^ A combination of centromeric and whole chromosome painting probes.

^‡^When present in the form of a homogeneous staining region or double minutes.

Abbreviations: CGH, comparative genomic hybridization; FFPE, formalin-fixed paraffin-embedded; FISH, fluorescence *in situ* hybridization; kb, kilobase; Mb, megabase; M-FISH, multicolor FISH; LOH, loss of heterozygosity; SKY, spectral karyotyping; SNP, single nucleotide polymorphism; OGM, optical genome mapping.

To detect translocations, two kinds of FISH probes are used, break-apart probes (BAPs) and double-color double fusion (DCDF) probes. The BAPs consist of differently labeled DNA probes, one complementary to sequences telomeric to and another centromeric to the breakpoint within the target gene. The DCDF probes contain two DNA probes labeled with different colors, one located at the breakpoint of one translocation partner and the second spanning the breakpoint in the other chromosome. The juxtaposition of both probes by the translocation creates a fusion signal with the third color (e.g., green and orange signals when juxtaposed make a yellow one). BAPs are less informative than DCDF probes in interphase cells because while they can show a break within the tested gene, they do not identify the translocation partner gene involved, although this can be visualized in metaphase cells. Moreover, because BAPs flank the locus of interest, small insertions might be undetected. Still, BAPs are useful for detection of clinically relevant translocations involving numerous partners of such promiscuous genes as *KMT2A* (formerly *MLL*), *MYC* or *BCL6*, and they are easier to evaluate because the separation of two signals is easy to recognize. However, since normal signals can sometimes be vaguely separated when BAPs are used, the normal signal pattern has to be carefully defined according to probe design. In contrast, a positive result with DCDF probes consists of two fusion signals, a result that is not likely to occur by coincidence.

Determination of cutoff values for the different probes used is important for accurate interpretation of FISH results. For DCDF probes, the cutoff is usually below 5 %, but it might be higher for some variant signal patterns, for instance, when one of the derivative chromosomes participating in the translocation is lost. For BAPs, whose signals are variable depending on the location of the breakpoints and probe design, the relative distance between the different color probes should be visually estimated in normal controls. A break is usually recognized if the distance between the two signals is at least twice the estimated signal diameter. Preferably, the BAP cutoff should be between 1 % and 5 %, although it might be higher, depending on the locus investigated and probe design.

Probe sets for the detection of most rearrangements associated with specific subtypes of leukemia or lymphoma are now available commercially and routinely used in cytogenetic laboratories to establish the diagnosis, select therapy, and monitor the results of therapy. However, it is important to underscore that in contrast to chromosome analysis, which allows the simultaneous recognition of all microscopically detectable aberrations in tumor cells, irrespective of whether they are numerical or structural, balanced or unbalanced, FISH can only be used to confirm or refute the presence of specific abnormalities that the probes used are designed to identify.

**Figure 1:**
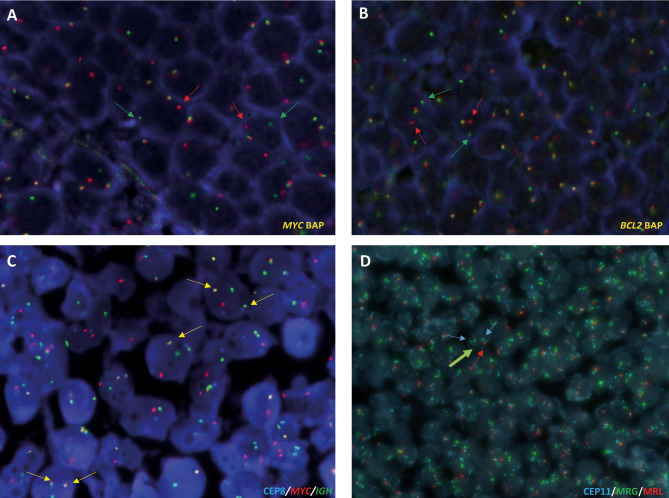
Fluorescence *in situ* hybridization (FISH) analysis of 14q32 (IGH)-associated translocations and 11q aberration in B-cell lymphomas on formalin-fixed paraffin-embedded (FFPE) tissues. **A and B**, Analysis of *MYC* and *BCL2* breaks using break apart probes (Metasystems) on FFPE sections of a Burkitt lymphoma (**A**) and follicular lymphoma cases (**B**). Breaks are indicated with green and red arrows **C**, FISH analysis using dual color dual-fusion *IGH*::*MYC* (Metasystems) on FFPE sections from a Burkitt lymphoma case. Fusions are indicated with yellow arrows. **D**, 11q aberration (Zytolight) identified in a high-grade B-cell lymphoma with 11q aberration case. The two centromeres of chromosome 11 are indicated with blue arrows, green arrows indicate 11q23.3 amplification (MRG, minimal region of gain) and red arrow highlights the presence of only one copy of 11q24.3 (MRL, minimal region of loss).

In contrast to FISH analyses performed on cytogenetic preparations, FFPE or frozen tissue sections can be difficult to work with and require further standardization techniques. Low efficiency of hybridization resulting in loss of signals and high background autofluorescence can lead to atypical signal patterns, making interpretation of results difficult. Nonetheless, FISH protocols have been successfully adapted for routine diagnosis (Figure 1). [17] Detecting genomic losses by FISH in FFPE tissues may be unreliable because part of the cell can be lost during the sectioning process, leading to false-positive results. Hence, for detection of deletions, the cutoff values for calling the case positive must be established at higher levels and proper negative controls have to be assessed. [17] As a result, commercial FISH probes designed for evaluation of losses usually include an internal FISH control labeled with a different fluorochrome that hybridizes to the centromere of the chromosome containing the locus of interest, or to a distal region of the same chromosome expected to be preserved if the deletion occurs. To investigate deletions, the evaluation by two different observers is recommended, as it is for the detection of translocations. Notably, the “typical” signal patterns might not be present in a given case; instead, one of the multitude of variant signal patterns caused by the complexity of the karyotype can be found. Evaluation and cut-offs have to be sensitive to such variant signal patterns. Moreover, in cases with low tumor cell content or partial infiltration, a prior or parallel morphological histological review and potentially selection of the area of interest can be useful to ensure an informative FISH testing on FFPE tissues. Non-tumor cell containing areas should also be screened to rule out germline alterations.

FISH can also be combined with immunophenotyping, which is especially useful in recognizing the cell lineage of a cytogenetically abnormal neoplastic clone. Simultaneous fluorescence immunophenotyping (FICTION technique) allows visualization of antigen expression of cells with chromosomal abnormalities thus directly correlating phenotypic and genotypic features of these cells. [18]

## Multicolor FISH techniques

SKY and M-FISH allow the simultaneous visualization of 22 pairs of autosomal chromosomes and sex chromosomes in different colors. Probes used for multicolor hybridizations are made using polymerase chain reaction (PCR)-amplification of flow-sorted chromosome libraries labeled with one to five fluorochromes that creates a unique color for each chromosome pair. In SKY, image acquisition is based on epifluorescence microscopy, charge-coupled device imaging and Fourier spectroscopy. [19] In M-FISH, separate images for each of five fluorochromes are taken with narrow band-pass microscope filters and the images are merged by dedicated software. Both techniques enable precise characterization of complex chromosome alterations, uncovering the composition of marker chromosomes, and identification of cryptic translocations and hidden amplification of chromosomal material. [19–21] Also, accurate description of pericentric and paracentric inversions can be achieved using the mBAND technique that creates multicolor banding pattern by application of differently labeled, overlapping probes from microdissected libraries of a single chromosome.

Multicolor images of metaphase cells are analyzed together with electronically inverted and contrast-enhanced DAPI images producing G-banding–like patterns that enable specific breakpoint assignments both in inter- and intrachromosomal alterations. The definitive recognition of chromosome aberrations and breakpoint assignment in structural aberrations is based on both spectral classification and G-banding. Additional FISH tests are often necessary to clarify ambiguous results, and/or confirm or refute the presumed involvement of specific genes located near breakpoints in structural alterations. The resolution of SKY/M-FISH for the detection of interchromosomal rearrangements is between 500 and 2000 kb and depends on the quality of the metaphase preparations. Akin to banding techniques, subtle, subtelomeric rearrangements cannot be detected by multicolor FISH methods.

## Comparative genomic hybridization

CGH allows scanning the entire genome for gains, losses and amplifications. [22] The test (tumor) and reference (normal) DNAs are differentially labeled and co-hybridized to either normal metaphase spreads (chromosomal CGH) or microarrays (array CGH). The advantage of CGH is that it requires only tumor DNA obtained from fresh or archived material and the reference DNA does not have to be from the same patient. The tumor DNA is usually labeled with a green fluorochrome (FITC/spectrum-green) and the reference DNA with a red fluorochrome (TRITC/spectrum-red). The differences in copy numbers between the tumor and normal DNA are reflected by differences in green and red fluorescence along the length of the chromosome. Limitations of CGH include its inability to uncover balanced abnormalities, a requirement for reliable detection that a loss or gain must be present in ~35 % of tumor cells, and that altered regions should be ≥10 Mb in size, whereas for high-level amplifications, the size of an amplicon cannot be <2 Mb. Importantly, no strict border exists to delineate low-copy gains from high-level amplifications in any of the technologies described. The overexpression of an oncogene that relates to the level of gain is usually more pronounced in focal alterations.

## Array-based copy number determination

Like CGH, CGH arrays rely on the difference in the copy numbers (CN) between differentially labeled test and reference DNA. The spots on the array are either DNA isolated from bacterial artificial chromosomes (BACs) containing human genomic DNA or oligonucleotides synthesized directly on the glass slide. The DNAs are directly labeled with fluorescent dyes, with display tumor DNA pseudo-colored red and reference DNA green, and a scanner detects the ratios of the fluorescence intensities of both dyes at each spot. High-density oligonucleotide arrays can detect somatic gains and losses of fewer than 5 kb, whereas germline variants can be excluded by application of paired germline DNA from the same individual. However, the CGH arrays do not detect regions of homozygosity.

In contrast, diploid stretches of homozygosity and heterozygosity can be recognized by genome-wide SNP arrays, which rely on oligonucleotide probes corresponding to the allelic variants of selected SNPs covering the whole genome. Heterozygosity is found when test DNA hybridizes to both probe variants, and homozygosity when the signal for only one allele is observed. Application of SNP arrays identified acquired copy neutral loss of heterozygosity (CN-LOH), which leads to loss of a normal allele and duplication of the mutated allele and thus can be functionally similar to a homozygous mutation, to be recurrent oncogenic events in both lymphomas and AML. The CN-LOH regions in lymphomas usually contain the *TP53* or *TNFRSF14* tumor suppressor genes, [23] whilst in AML, CN-LOH involves loci of genes frequently mutated in this leukemia and leads to homozygous mutations of *FLT3*, *RUNX1*, *CEBPA* and *WT1*. [24]

## Optical genome mapping (OGM)

OGM is a robust technology capable of detecting numerical and structural, both unbalanced and balanced, chromosome alterations in a single step. [25, 26] OGM relies on high-throughput imaging of high molecular weight DNA (>250 kb) labeled fluorescently at a specific 6 bp sequence motif that occurs 15 times per 100 kb in the human genome. The unique labeling pattern enables identification of the genomic location of every molecule, producing a consensus map that can be compared to a reference genome. Structural variants are detected from single molecules, from 500 bp resolution and down to 1 % allele fraction. Also, genome coverage depth information is used to detect copy number alterations and chromosome gains and losses. OGM does not require cell culture before sample processing and results may be obtained in 4 to 6 days. Recent OGM studies analyzed specimens from patients with ALL, AML, MDS and CLL. It has been proposed that OGM could replace cytogenetic assays as a tool for analysis of hematologic malignancies. [25, 26] However, because OGM uses the CN variation (CNV)-pipeline, employing quantification of uniquely attributable genetic material to detect variants, the detection of ploidy changes such as triploidy and higher-order polyploidy is difficult. Also, OGM requires a significant amount of fresh and/or frozen blood cells whereas previously extracted DNA is not suitable for analysis.

## Technologies for detection of single nucleotide variants (SNVs) in leukemia- and lymphoma-associated genes

In addition to cytogenetic analyses, current genetic evaluation of patients with hematologic neoplasms at diagnosis includes testing of several genes for SNVs, which have been demonstrated to have clinical relevance, such as, for example, *NPM1*, *FLT3*,* CEBPA*, *DNMT3A*, *TP53*, *RUNX1*, *IDH1/2*, *WT1*,* PAX5*,* CDKN2A* or *NRAS*. [27, 28] This does not only include somatic alterations but also germline (panel) testing in the case of, for example, myeloid neoplasms or immunodeficiency-associated lymphomas associated with germline predisposition or inborn errors of immunity, respectively. Special consideration in many diagnostic processes is on the detection and sequencing analysis of the clonally rearranged immunoglobulin (IG) and T-cell receptor (TR) loci. Several molecular methodologies are available for both candidate gene and clonal IG/TR receptor gene analyses, including PCR-based Sanger sequencing, reverse transcription PCR (RT-PCR) and high-throughput next-generation sequencing (NGS) of targeted, gene-specific panels. [27, 28] Moreover, whole genome sequencing (WGS) analyses ranging from shallow to full deep genomic sequencing and short-read to long-read analyses can also provide an overall, high-resolution karyotype-like overview of genomic alterations. [29, 30] A potential clinical utility of WGS in AML was shown recently, and WGS was suggested to be an alternative to cytogenetic and targeted sequencing assays. [29] However, an in-depth review of WGS, OGM and other novel molecular methods, including their technical (e.g., DNA quantity, quality and tumor cell content), logistic, financial and clinical aspects, concluded that the instant implementation of these technologies by clinical laboratories worldwide is rather unlikely. [30]

## What to use in the daily practice?

Not all technologies described above are widely used in the daily practice and several of them are mainly applied in translational setting. In the case of liquid samples like in leukemias and with some correlation to their differentiation stage, a combination of chromosome banding (in more immature malignancies), array-based copy number profiling, FISH and targeted (panel) sequencing is mostly widely applied. This might be complemented with RNA-based fusion gene analysis be it targeted or unbiased. In the case of solid tumoral presentations and particularly FFPE tissues, FISH, array-based copy number profiling and targeted (panel) sequencing are the methods widely used. As outlined above, the WHO-HAEM5 does not recommend specific technologies, and as the classification has to be applicable worldwide, also in low- and middle-resource countries, genetic alterations that can be targeted by standard technologies are included in the classification. In the translational setting as in clinical trials, exome-sequencing, WGS and RNAseq are increasingly applied, but standardization is pending in many instances.

## Other nucleic acid-based technologies

Gene-expression profiling using RNA-based methods like array-based technologies or RNAseq, aside from the detection of fusion transcripts, does not (yet) play a major role in WHO-HAEM5. It can be applied, for example, to detect a subset of ALL with *BCR*::*ABL*-like features or to differentiate germinal-center B-cell-like from activated B-cell-like diffuse large B-cell lymphoma, but at least the latter distinction is more frequently based on immunohistochemical features in daily diagnostic work. Similarly, and in contrast to, e.g. the WHO-classification of brain tumors, DNA methylation profiling does not have (yet) any diagnostic importance in the classification of hematologic neoplasms, though clinically relevant subsets of CLL can be identified.

## Conclusions

Recent years have seen a dramatic increase in research of hematologic neoplasms using NGS technologies, including WGS. However, WGS is primarily used in translational research carried out by academic centers. Although ongoing efforts are likely to enable successful application of NGS in the clinic in the future, for now, chromosome banding analysis, FISH and SNP arrays remain the gold standard of cytogenetic testing in hematologic neoplasms.
